# Unique Strain of *Borrelia miyamotoi* in *Ixodes pacificus* Ticks, California, USA

**DOI:** 10.3201/eid2212.152046

**Published:** 2016-12

**Authors:** Vanessa J. Cook, Natalia Fedorova, Warren P. Macdonald, Robert S. Lane, Alan G. Barbour

**Affiliations:** University of California, Irvine, California, USA (V.J. Cook, A.G. Barbour);; University of California, Berkeley, California, USA (N. Fedorova, R.S. Lane);; San Mateo County Mosquito and Vector Control District, Burlingame, California, USA (W.P. Macdonald)

**Keywords:** vector-borne infections, ticks, Ixodes pacificus, Borrelia miyamotoi, California, zoonoses, bacteria, United States

**To the Editor:** *Borrelia miyamotoi* causes a recently recognized tickborne zoonosis in Eurasia and North America (*1*). The species has been detected in *Ixodes persulcatus* ticks in Asia and Russia, *I. ricinus* ticks in Europe, and *I. scapularis* and *I. pacificus* ticks in North America. In most of these regions, *B. miyamotoi* is sympatric with Lyme disease agents, such as *B. burgdorferi*, and both pathogens are transmitted locally by the same species of *Ixodes* ticks. *B. miyamotoi* generally is less prevalent than *B. burgdorferi* in nymphs and adults in North America (*2*), except in California, where the prevalences of the 2 species in populations of nymphal and adult *I. pacificus* ticks are similar (*3*–*6*).

Genomes of isolates of *B. miyamotoi* from *I. persulcatus* and *I. scapularis* ticks have been sequenced (*7*). Comparatively less was known about *B. miyamotoi* in *I. pacificus* ticks. Limited sequence data of 16S ribosomal RNA and flagellin genes and the 16S-23S intergenic spacer (IGS) were sufficient to identify the *I. pacificus*–borne spirochete as a sister taxon to *B. miyamotoi* from elsewhere (*3*,*4*). Until *B. miyamotoi* is isolated from *I. pacificus* ticks, determination of additional sequences from *I. pacficus* ticks from California addresses 2 issues of phylogeographic and potential epidemiologic importance: Is the California population of *B. miyamotoi* more akin to the strain across the Pacific Rim or to the strain thousands of kilometers to the east in North America? Will the noted pattern of exclusive association between the genotype of *B. miyamotoi* and the species of *Ixodes* vector continue to hold (*1*)?

We evaluated DNA extracts of *B. miyamotoi*–infected *I. pacificus* ticks collected by and stored at 2 laboratories in the San Francisco Bay area of California. Ticks had been collected while questing either on low vegetation or in leaf litter. To confirm *B. miyamotoi* in candidate extracts and to exclude extracts that also contained *B. burgdorferi* sensu lato, we used a quantitative PCR, which differentiates relapsing fever and Lyme disease group species (*2*). Two extracts that met these criteria were Sonom53 from a nymph in Sonoma County, California (38.328758, −122.625286), and SMA107 from an adult male tick in San Mateo County, California (37.466999, −122.283532). We amplified DNA by PCR for 1,307 bp of the 16S ribosomal RNA gene (*8*) and variable lengths of the IGS (*9*). In addition, we performed PCR amplification and sequencing of partial sequences of 8 chromosomal genes used for multilocus sequence typing (MLST): *clpA*, *clpX*, *nifS*, *pepX*, *pyrG*, *recG*, *rplB*, and *uvrA* (*10*). The primers (and annealing temperatures for 35 cycles) were as given (http://pubmlst.org/borrelia), except for these modifications: *clpA* (53°C); *clpX* forward 5′-CCGTTGCTATTTGTTTTGAATGCTCT-3′ (55°C); *pepX* forward 5′-TTAAAACTTGATGATAAATGGTCATTA-3′ and reverse 5′-TTAAAACTTGATGATAAATGGTCATTA-3′ (52°C); *pyrG* forward 5′-CTTTTAGTAATTGAGATTGGTGGT-3′ and reverse 5′-CAGCATCAAGTATTCCACAAAC-3′ (55°C); *recG* forward 5′-CTAGCATTCCTTTAGTTGAGGC-3′ and reverse 5′-TTSTGTTAAAGGTTCCTTATAAAG-3′ (52°C); *rplB* forward 5′-ATTAAAACTTATAGGCCAAAAAC-3′ and reverse 5′-GGCTGACCCCAAGGAGAT-3′ (55°C); and *uvrA* forward 5′-GCTTAAATTTTTAATTGATGTTGGA-3′ and reverse 5′-CAAGGAACAAAAATRTCAGGC-3′ (52°C). On a Bio-Rad T100 thermal cycler (Hercules, CA, USA) and with Apex Master mix (Genesee Scientific, San Diego, CA, USA), PCR extension at 72°C was 1.5 min for *clpX* and 1.0 min for others; final elongation was for 5 min at 72°C. Products were sequenced over both strands at GENEWIZ (San Diego, CA, USA) by the Sanger method either directly or after cloning into a plasmid vector. Resultant sequences were aligned with homologous sequences (Figure). Alignments and distance neighbor-joining and maximum-likelihood phylograms were generated with Seaview4 (http://doua.prabi.fr/software/seaview). The equal length MLST sequences, as specified (*10*), for each locus were concatenated.

**Figure:**
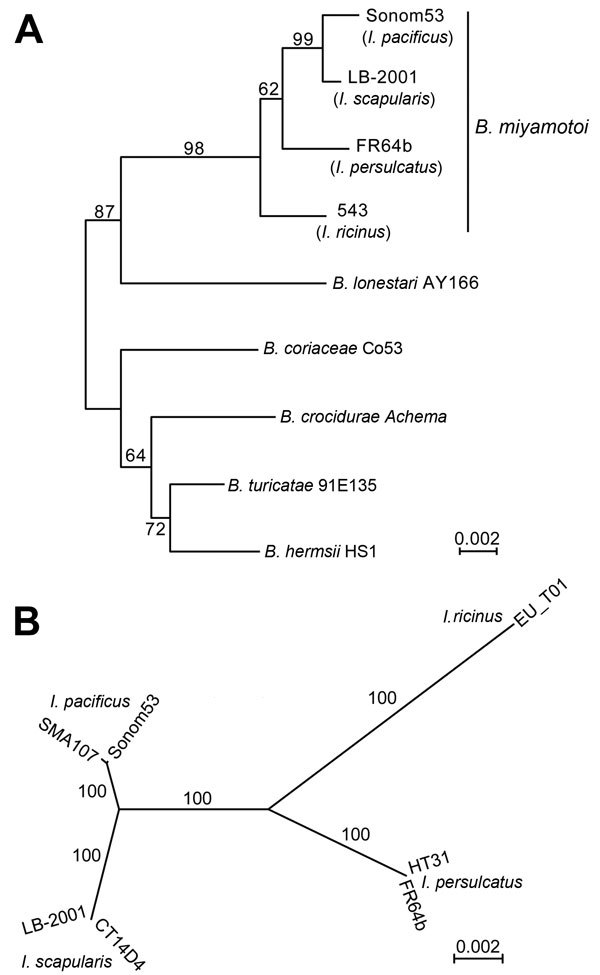
Phylograms of 16S ribosomal RNA sequences (A) and of multilocus sequence typing (MLST) genes (B) of *Borrelia miyamotoi* strains from *Ixodes* ticks collected in California, USA, and selected other *Borrelia* species. A) Rooted neighbor-joining distance phylogram of observed differences. Percentage support for clades was evaluated by 1,000 bootstrap replications, and values are indicated along branches. The GenBank accession number for the partial 16S ribosomal RNA gene of Sonom53 is KU196080. GenBank accession numbers for 16S ribosomal RNA genes of other *B. miyamotoi* strains are NR_121757 (LB-2001), and KJ847049 (543), and AY604976 (FR64b). GenBank accession numbers for corresponding sequences of designated strains of other species are AY166715 for *B. lonestari* and, for the 4 species constituting the outgroup, AF210134 for *B. coriaceae*, GU350713 for *B. crocidurae*, NR_102958 for *B. turicatae*, and NR_102957 for *B. hermsii*. The tick species sources of the *B. miyamotoi* organisms are indicated. B) Unrooted maximum-likelihood phylogram for 8 concatenated, codon-aligned MLST genes. The model of nucleotide substitution was HKY85 and the empirically estimated γ shape parameter was 0.01. Percentage support for clades was evaluated by 100 bootstrap replications by using full-heuristic search, and values are indicated along branches. GenBank accession numbers for Sonom53 partial sequences of *clpA*, *clpX*, *nifS*, *pepX*, *pyrG*, *recG*, *rplB*, and *uvrA* genes are KU23498–KU234405. The partial sequence of SMA107 *pyrG* is KU307254. The corresponding sequences for FR64b, LB-2001, and CT14D4 were obtained from the complete chromosomes (GenBank accession nos. CP004217, CP006647, and CP010308, respectively). The MLST sequences for strains EU-T01 and HT31 were obtained from the Borrelia MLST Database (http://pubmlst.org/borrelia/), where they have identification numbers of 1279 and 1275, respectively. Scale bar indicates nucleotide substitutions per site.

We determined a rooted neighbor-joining phylogram of 16S ribosomal RNA gene sequences of *B. miyamotoi* from different *Ixodes* species and that of *Amblyomma americanum* tickborne *B. lonestari* (Figure, panel A). Other species of the relapsing fever group served as an outgroup. *B. miyamotoi* sequences from *I. pacificus* ticks in 2 San Francisco Bay area counties clustered with sequences from *I. scapularis*–borne organisms rather than with *I. persulcatus*–borne organisms in Asia or an *I. ricinus*–borne isolate in Europe. This analysis confirmed that the organism in *I. pacificus* was *B. miyamotoi*. An unrooted phylogram of 4,776 nt of concatenated MLST sequences originating in *I. pacificus*, *I. scapularis*, *I. persulcatus*, or *I. ricinus* ticks had similar topology and differentiated the different strains (Figure, panel B). The *B. miyamotoi* organisms from 2 counties differed at 1 position, a synonymous transition in *pyrG*, among the MLST loci. IGS sequences of the 2 organisms were the same (GenBank accession no. KU184505) and identical to the IGS of other *B. miyamotoi* in *I. pacificus* ticks (e.g., GenBank accession no. KF957669). As observed previously (*4*,*9*), they were distinct from strains associated with other *Ixodes* species.

In conclusion, we identified differences in several genetic loci between *B. miyamotoi* in *I. pacificus* ticks and organism strains associated with other *Ixodes* species. However, we found a close phylogenetic relationship between organisms from the far-western and the northeastern United States.

## References

[1] Krause PJ, Fish D, Narasimhan S, Barbour AG. *Borrelia miyamotoi* infection in nature and in humans. Clin Microbiol Infect. 2015;21:631–9. 10.1016/j.cmi.2015.02.006 25700888 PMC4470780

[2] Barbour AG, Bunikis J, Travinsky B, Hoen AG, Diuk-Wasser MA, Fish D, et al. Niche partitioning of *Borrelia burgdorferi* and *Borrelia miyamotoi* in the same tick vector and mammalian reservoir species. Am J Trop Med Hyg. 2009;81:1120–31. 10.4269/ajtmh.2009.09-0208 19996447 PMC2841027

[3] Mun J, Eisen RJ, Eisen L, Lane RS. Detection of a *Borrelia miyamotoi* sensu lato relapsing-fever group spirochete from *Ixodes pacificus* in California. J Med Entomol. 2006;43:120–3. 10.1603/0022-2585(2006)043[0120:DOABMS]2.0.CO;2 16506458

[4] Fedorova N, Kleinjan JE, James D, Hui LT, Peeters H, Lane RS. Remarkable diversity of tick or mammalian-associated borreliae in the metropolitan San Francisco Bay area, California. Ticks Tick Borne Dis. 2014;5:951–61. 10.1016/j.ttbdis.2014.07.015 25129859

[5] Padgett K, Bonilla D, Kjemtrup A, Vilcins IM, Yoshimizu MH, Hui L, et al. Large scale spatial risk and comparative prevalence of *Borrelia miyamotoi* and *Borrelia burgdorferi* sensu lato in *Ixodes pacificus.* PLoS One. 2014;9:e110853. 10.1371/journal.pone.0110853 25333277 PMC4205013

[6] Salkeld DJ, Nieto NC, Carbajales-Dale P, Carbajales-Dale M, Cinkovich SS, Lambin EF. Disease risk and landscape attributes of tick-borne *Borrelia* pathogens in the San Francisco Bay area, California. PLoS One. 2015;10:e0134812. 10.1371/journal.pone.0134812 26288371 PMC4545583

[7] Barbour AG. Phylogeny of a relapsing fever *Borrelia* species transmitted by the hard tick *Ixodes scapularis.* Infect Genet Evol. 2014;27:551–8. 10.1016/j.meegid.2014.04.022 24813576 PMC4182126

[8] Barbour AG, Maupin GO, Teltow GJ, Carter CJ, Piesman J. Identification of an uncultivable *Borrelia* species in the hard tick *Amblyomma americanum*: possible agent of a Lyme disease–like illness. J Infect Dis. 1996;173:403–9. 10.1093/infdis/173.2.403 8568302

[9] Bunikis J, Tsao J, Garpmo U, Berglund J, Fish D, Barbour AG. Typing of *Borrelia* relapsing fever group strains. Emerg Infect Dis. 2004;10:1661–4. 10.3201/eid1009.040236 15498172 PMC3320305

[10] Margos G, Gatewood AG, Aanensen DM, Hanincova K, Terekhova D, Vollmer SA, et al. MLST of housekeeping genes captures geographic population structure and suggests a European origin of *Borrelia burgdorferi.* Proc Natl Acad Sci U S A. 2008;105:8730–5. 10.1073/pnas.0800323105 18574151 PMC2435589

